# Factors associated with approaching Pilot Peer Support: a cross-sectional study

**DOI:** 10.1093/occmed/kqae033

**Published:** 2024-06-10

**Authors:** B Bråstad, R Jonsäll-Harris, M Melin, F Folke

**Affiliations:** Department of Clinical Neuroscience, Karolinska Institute, 171 65 Stockholm, Sweden; Department of Clinical Neuroscience, Karolinska Institute, 171 65 Stockholm, Sweden; Department of Clinical Neuroscience, Karolinska Institute, 171 65 Stockholm, Sweden; Department of Clinical Neuroscience, Karolinska Institute, 171 65 Stockholm, Sweden

## Abstract

**Background:**

Pilot Peer Support Programs (PPSP) have been introduced in Europe as a measure to facilitate commercial pilots’ mental health help-seeking in a confidential and non-punitive manner. However, research is scarce regarding what promotes and prevents pilots from approaching PPSP.

**Aims:**

To investigate if, and in which way, different organizational and individual factors are associated with pilots’ attitudes towards approaching PPSP, and to examine the prevalence of possible cases of depression and anxiety disorders among commercial pilots in Europe.

**Methods:**

Data were collected using an anonymous web-based survey (*n* = 4494), covering pilots’ work conditions, health and flight safety. Logistic regression was used to determine the impact of objective and psychosocial work environment factors, mental health factors, and demographic factors.

**Results:**

Key findings were that just culture (odds ratio [OR] = 2.65, 95% confidence interval [CI] 1.97, 3.56), type of employment (OR = 0.60, 95% CI 0.46, 0.78), minimum guaranteed pay (OR = 1.98, 95% CI 1.48, 2.65), and symptoms of depression (OR = 0.62, 95% CI 0.50, 0.76) and anxiety (OR = 0.66, 95% CI 0.54, 0.80) significantly predicted pilots’ attitude towards approaching PPSP. The prevalence of pilots scoring above threshold for possible depression (18%) and anxiety disorders (23%) were determined.

**Conclusions:**

Pilot Peer Support in its current form appears to be an insufficient means to facilitate pilots’ mental health help-seeking, but could have an important preventive purpose. The findings could assist authorities and operators in developing measures to facilitate pilots’ help-seeking, and improve mental health and flight safety.

## Teaser

Pilot Peer Support Programs (PPSP) are mandatory in Europe to facilitate pilots’ mental health help-seeking. However, little is known about which factors promote and prevent pilots from approaching PPSP. A survey of European pilots shows that those reporting a just culture, support from colleagues and management, a fixed monthly salary and direct employment were more inclined to approach PPSP. Pilots with clinically relevant symptoms of depression and anxiety disorders were less inclined to approach PPSP.

**Key learning points**

**What is already known about this subject:**

- Depression and anxiety among pilots could have a great impact on flight safety as it might impair performance.
- Recognizing depression among pilots is, however, known to be challenging, and there is a probable underreporting of mental health issues among commercial pilots.
- As a measure to prevent mental health issues and facilitate pilots’ help-seeking, Pilot Peer Support Programs must be available for all commercial pilots in Europe, but research is scarce regarding what promotes and prevents pilots from approaching Pilot Peer Support Programs.

**What this study adds:**

- The study provides new, concrete insights into which factors promote and prevent pilots from approaching Pilot Peer Support Programs.
- Contextual factors such as direct employment, a minimum guaranteed pay, just culture, and support from colleagues and managers appear to have a positive influence on pilots’ inclination to approach Pilot Peer Support Programs.
- Symptoms of depression and anxiety, on the other hand, represent significant barriers to approaching Pilot Peer Support Programs.

**What impact this may have on practice or policy:**

- The identification of organizational and individual factors that promote or prevent pilots from approaching peer support can assist airline operators and aviation authorities in developing measures and regulations that facilitate pilots’ help-seeking, in turn, improving mental health and flight safety.
- In order to promote pilots’ inclination to approach PPSP, operators and authorities could develop and enhance good practices and policies regarding atypical employment contracts and guaranteed minimum pay.
- In addition, it could be important for operators to routinely measure how pilots perceive their psychosocial work environment, as psychosocial work environment factors appear to influence pilots’ mental health help-seeking.

## Introduction

Depression and anxiety are among the leading causes of disability worldwide, yet a minority of affected individuals ever seek help [1]. There is reason to believe that commercial pilots are particularly reluctant to self-disclose or seek help for mental health issues [2]. Despite requirements to report any decrease in medical fitness to an Aero-Medical Examiner, pilots’ disclosure of mental health issues generally implies the revocation of their medical certification, temporarily disqualifying them from flight duties [3]. Research on the prevalence of depression and anxiety among pilots is scarce, but data from an anonymous web-based survey found that 13% may be suffering from clinically relevant levels of depression [4]. Together with stigma, the potential financial and professional consequences associated with poor mental health likely cause underreporting of mental health issues among commercial pilots [2]. The tragic accident of Germanwings Flight 9525 serves as an extreme example of the threats that undisclosed mental health issues pose to the aviation industry. Having been diagnosed with a severe psychiatric disorder just months earlier, the first officer intentionally brought the aircraft, including its 150 occupants, into the French Alps. Despite obligations to report any decrease in medical fitness, the first officer had refrained from doing so [5,6]. The accident called for immediate attention to pilots’ mental health and its impact on flight safety [5,7].

Measures to facilitate mental health help-seeking among pilots include requirements that all commercial pilots working in Europe have access to Pilot Peer Support Programs (PPSP). PPSP consist of pilot peers who are trained and supervised by mental health professionals, with the purpose of supporting pilots in recognizing, coping with and overcoming any problems that potentially impact their ability to safely conduct a flight [8]. The objective of PPSP slightly differs from that of peer support programmes traditionally used in other industries, in that prevention, rather than post-trauma support, is the main focus [9]. PPSP enables means to access help and support in a non-punitive manner, whilst maintaining confidentiality towards the employer [10]. This allows pilots to disclose mental health issues without jeopardizing their employment status, in line with the just culture philosophy widely adopted within aviation safety. However, the organizational conditions in which peer support is utilized might not always be as ‘non-punitive’ as the regulation demands [7].

Commercial pilots’ work environment is characterized by high workload, irregular working hours, shift work and systematic disruptions of circadian rhythms, all having potential associations with the development of mood disorders [11]. Symptoms such as sleep disruptions, concentration difficulties, lack of energy, reduced motivation and suicidal thoughts may deteriorate pilots’ abilities to safely conduct flights [12,13]. Thereto, pilots’ terms of employment may constitute objective measures of job insecurity. Today, more than one out of five European pilots have atypical employment types, that is, not being directly employed by the airline or not having a permanent contract [14]. Furthermore, many pilots lack income loss insurance and the preferential right to alternative employment if becoming medically unfit, adding to their vulnerability [7]. The extent to which such work conditions are associated with pilots’ mental health help-seeking, in particular to PPSP, are yet to be discovered.

PPSP are primarily preventive measures and require active help-seeking from the pilots themselves. Therefore, it is important to investigate what promotes and prevents pilots from contacting peer support. The aim of this study is to investigate if, and in which way, different organizational and individual factors are associated with attitudes towards approaching PPSP, and to examine the prevalence of possible cases of depression and anxiety disorders among commercial pilots in Europe.

## Methods

The present study used cross-sectional data from a research project at Karolinska Institute in Sweden, investigating the changing landscape of the aviation industry, including the impact of prior deregulation and the current challenges posed by the coronavirus disease 2019 (COVID-19) pandemic. Collection of data began in April 2022 and continued until September 2022. The projects had a priori ethical approvals from the Ethics Review Authority in Sweden (Dnr2016/250-31/2 and 2021-05750-02).

The participants were commercial pilots working in Europe, recruited with the assistance of the European Cockpit Association (ECA) and advertised on social media. ECA, an umbrella organization representing national pilot unions with more than 40 000 pilots across 36 states, was informed about the participation rates across the European nations, and increased promotional activities in countries with low participation [15]. Contacted pilots were invited to complete an anonymous web-based survey about their work environment and health. To be eligible to participate, pilots had to be currently working at the time of the data collection. For inclusion in this study, participants also had to complete the survey to the closing demographic questions. All pilots were informed about the aim of the survey, rights to withdraw, confidentiality and contact details to the project leader. All pilots provided their informed consent prior to participation. The survey included unidentified yet sensitive personal data such as physical and mental health and trade union membership. Participation was not expected to cause any adverse reactions or discomfort to the participants.

The survey covered employment and work conditions, physical and mental health, flight safety, as well as the impact of the COVID-19 pandemic on these areas. The survey consisted of well-reputed questions and new questions designed to fit the purpose of the project. A reference group made up of pilots was used to review and suggest changes to the survey. For the present study, relevant survey questions were selected based on previously established associations found in the literature on help-seeking and peer support, and grouped into organizational factors and individual factors. The organizational factors were further divided into psychosocial and objective work environment factors, and the individual factors were divided into mental health and demographic factors.

Table 1 illustrates a summary of all variables, including the survey questions on which they were based, participants’ response options and any variable recoding, including references where applicable. Survey questions without references were created by the research group. The mental health factors included the variables depression and anxiety, as measured by the Hospital Anxiety and Depression Scale (HADS) developed by Zigmond and Snaith [25]. HADS has been shown to perform well in the general population [26] and in the working population [27]. HADS has two subscales, one for depression (HADS-D) and one for anxiety (HADS-A), consisting of seven items each with responses on a four-point Likert scale. Scores were dichotomized using a cut-off value of 8 on the respective subscales of HADS. The cut-off was selected in order to provide an optimal balance between specificity and sensitivity in screening for depression and anxiety disorders [26], in line with what Zigmond and Snaith proposed in their original paper [25].

**Table 1.** Summary of the variables in the study, the survey questions on which the variables are based, the response options and any recoding

Variable category	Variable (α)	Survey question(s)/statement(s)^a^	Response options	Variable recoding^b^
Dependent variable	Approaching peer support	‘If needed, would you approach Peer Support Service?’	Yes/No	–
Psychosocial work environment factors	Job insecurity (α = 0.85)	‘I worry about losing my job’ [16]	(1) to (5)	Low (1–2)/Medium (3)/High (4–5)
		‘I worry about the future stability of my employment’	(1) to (5)	
	Trust in management	‘In general, employees trust management’ [17]	(1) to (5)	Low (1–2)/Medium (3)/High (4–5)
	Disclosure to employer	‘If you were feeling depressed or anxious, would you talk to your employer about this?’ [18]	Yes/No	–
	Support from colleagues	‘If needed my colleagues help and support me in my work’ [17]	(1) to (5)	Low (1–2)/Medium (3)/High (4–5)
	Support from manager	‘If needed my manager helps and supports me in my work’ [17]	(1) to (5)	Low (1–2)/Medium (3)/High (4–5)
	Just culture (α = 0.79)	‘We get timely feedback on the safety issues we raise’ [19, 20]	(1) to (5)	Low (1–2)/Medium (3)/High (4–5)
		‘I get adequate feedback from my airline if I have questions or raise suggestions’ [21, 22]	(1) to (5)	
		‘I feel comfortable in and supported when using my authority to make safety-related decisions’	(1) to (5)	
	Workplace satisfaction	‘In general, how satisfied are you with your employer?’ [23]	(1) to (5)	Low (1–2)/Medium (3)/High (4–5)
Objective work environment factors	Type of employment	‘What is your relationship with the airline you currently work for?’ [24]	Direct employment Employee with agency Self-employed Pay-to-fly Other	Typical (Direct emp. and Permanent contr.) / Atypical (All other combinations)
		‘What kind of employment contract do you have?’ [24]	Permanent contract Temporary or fixed-term contract Other	
	Minimum guaranteed pay	‘Do you get a minimum guaranteed pay regardless of the hours you have flown?’	Yes/No	–
Mental health factors	Depression (α = 0.83)	HADS-D [25]	(1) to (4)	<8/≥8 (score on HADS-D)
	Anxiety (α = 0.83)	HADS-A [25]	(1) to (4)	<8/≥8 (Score on HADS-A)
Demographic factors	Age	‘Age…’	Continuous	≤35/36-45/46–55/≥56
	Gender	‘Are you…?’	Male/Female/Other	–
	Relationship status	‘What is your relationship status?’	Single Married/Civil Union/Living with partner In a relationship living apart	Single/relationship (all other responses)

α = Cronbach’s alpha for variables created as an index of multiple Likert-type survey questions/statements.

^a^Questions/statements without references are created by the research group.

^b^Recoding into dummy variables for ease of interpretation and to increase category sizes. Variables indicated with ‘–’ retained the original formatting and were not recoded.

Statistical analyses on the survey data were performed using IBM SPSS Statistics Version 28 for Mac OS. Data were analysed using descriptive statistics and logistic regression. Throughout the statistical analyses, an alpha level of 0.05 was applied. Missing data were eliminated using list-wise deletion. Logistic regression was used to calculate the independent variables’ impact on approaching PPSP, while controlling for the influence of all other independent variables. Unadjusted logistic regression models were constructed to measure the crude estimates of approaching PPSP. In the adjusted model, the independent variables were analysed simultaneously with regard to their adjusted impact on approaching PPSP. This model served to satisfy the aim of providing a broad overview of how organizational and individual factors are associated with approaching PPSP. The independent variables were checked for multicollinearity using Spearman’s rank correlation coefficients. A cut-off was set at *ρ* > 0.7, as it is a commonly used cut-off for multicollinearity across scientific disciplines [27]. One psychosocial work environment factor was identified as having problems with collinearity and therefore excluded from further analysis.

## Results

The survey was commenced by 7168 pilots with a completion rate of 63%, leaving 4494 pilots (95% male) in the final sample. Missing data did not exceed 3% in the unadjusted models. Missing data were 6% in the adjusted model. The average age of the pilots was 43.9 years (standard deviation [SD] = 9.6, *Md *= 44). Among the pilots, 4036 (90%) reported being in a relationship and 439 (10%) reported being single. More than 90% of the pilots were engaged in scheduled passenger services or freight transport services. The remainder was engaged in non-scheduled passenger services, business aviation services and 3% responded ‘Other’. Of the participants, 69% responded that they would approach the PPSP if needed and 36% would talk to their employer if feeling depressed or anxious. In the sample, 18% of the participants scored 8 or higher on HADS-D, and 23% scored 8 or higher on HADS-A, that is, above the threshold for possible depression or anxiety disorder [28].

Table 2 illustrates the unadjusted and adjusted effects of the organizational and individual factors on approaching PPSP, using logistic regression. In the crude estimates, all psychosocial work environment factors significantly predicted approaching PPSP. For example, compared to pilots reporting just culture as ‘low’, those reporting ‘high’ just culture were more likely to approach PPSP (odds ratio [OR] = 8.80, 95% confidence interval [CI] 7.04, 11.01, *P* ≤ 0.001). Crude estimates of both objective work environment factors were significant predictors, where pilots with typical employment types and minimum guaranteed pay were more inclined to approach PPSP. Regarding the individual factors, symptoms of depression (OR = 0.28, 95% CI 0.24, 0.33, *P* ≤ 0.001) and anxiety (OR = 0.32, 95% CI 0.28, 0.37, *P* ≤ 0.001) were negatively associated to approaching PPSP in the unadjusted models. Relationship status (OR = 1.25, 95% CI 1.01, 1.53, *P* = 0.039) was a weak yet significant predictor of approaching PPSP, while age and gender were not.

**Table 2.** Unadjusted (Model 0) and adjusted effects (Model 1) of organizational and individual factors on approaching PPSP using logistic regression

		Model 0^a^			Model 1^b^^,^^c^^,^^d^		
Variable category	Independent variables	*B*	SE	OR 95% CI [LL, UL] *P*	*B*	SE	OR 95% CI [LL, UL] *P*
Psychosocial work environment factors	Job insecurity (*n* = 4461)						
	Low			1			1
	Medium	−0.28	0.09	0.76 [0.64, 0.90]^**^	0.19	0.10	1.21 [0.99, 1.49]
	High	−0.70	0.09	0.50 [0.42, 0.59]^***^	0.16	0.11	1.18 [0.96, 1.45]
	Trust in management (*n* = 4458)						
	Low			1			1
	Medium	0.86	0.10	2.35 [1.92, 2.88]^***^	−0.06	0.13	0.95 [0.73, 1.22]
	High	1.13	0.10	3.08 [2.52, 3.77]^***^	−0.23	0.14	0.79 [0.60, 1.05]
	Disclosure to employer (*n* = 4435)						
	No			1			1
	Yes	1.71	0.09	5.52 [4.66, 6.55]^***^	1.22	0.10	3.39 [2.80, 4.11]^***^
	Support from colleagues (*n* = 4459)						
	Low			1			1
	Medium	0.26	0.21	1.29 [0.87, 1.94]	-0.02	0.24	0.99 [0.62, 1.56]
	High	1.37	0.18	3.94 [2.75, 5.64]^***^	0.57	0.22	1.77 [1.16, 2.70]^**^
	Support from manager (*n* = 4457)						
	Low			1			1
	Medium	0.62	0.08	1.85 [1.58, 2.17]^***^	0.01	0.10	1.01 [0.83, 1.23]
	High	1.41	0.08	4.10 [3.50, 4.81]^***^	0.27	0.12	1.32 [1.05, 1.65]^*^
	Just culture (*n* = 4447)						
	Low			1			1
	Medium	1.21	0.11	3.35 [2.72, 4.11]^***^	0.62	0.12	1.87 [1.47, 2.38]^***^
	High	2.18	0.11	8.80 [7.04, 11.01]^***^	0.97	0.15	2.65 [1.97, 3.56]^***^
	Workplace satisfaction (*n* = 4459)						
	Low			1			1
	Medium	0.47	0.10	1.61 [1.32, 1.96]^***^	0.06	0.12	1.06 [0.84, 1.34]
	High	1.26	0.07	3.52 [3.05, 4.07]^***^	0.37	0.11	1.45 [1.18, 1.79]^***^
Objective work environment factors	Type of employment (*n* = 4455)						
	Typical			1			1
	Atypical	−0.98	0.11	0.38 [0.30, 0.47]^***^	−0.51	0.14	0.60 [0.46, 0.78]^***^
	Minimum guaranteed pay (*n* = 4448)						
	No			1			1
	Yes	1.02	0.12	2.78 [2.19, 3.53]^***^	0.68	0.15	1.98 [1.48, 2.65]^***^
Mental health factors	Depression (*n* = 4405)						
	<8			1			1
	≥8	−1.27	0.08	0.28 [0.24, 0.33]^***^	−0.48	0.11	0.62 [0.50, 0.76]^***^
	Anxiety (*n* = 4388)						
	<8			1			1
	≥8	−1.14	0.07	0.32 [0.28, 0.37]^***^	−0.42	0.10	0.66 [0.54, 0.80]^***^

*B* = unstandardized regression; SE = standard error; OR = odds ratio; CI = confidence interval; LL = lower limit; UL = upper limit.

^*^ *P* < 0.05; ^**^P < 0.01; ^***^P < 0.001.

^a^Model 0 = Unadjusted direct effects.

^b^Model 1 = Effects adjusted for all other factors in the model, including age, gender, and relationship status.

^c^ *n* = 4215.

^d^Nagelkerke = 0.267, −2 Log-likelihood = 4288.940.

Among the psychosocial work environment factors, job insecurity and trust in management no longer predicted approaching PPSP when adjusting for the other predictors. The remainder of the psychosocial work environment factors maintained significant associations in the adjusted model. For example, disclosure to employer (OR = 3.39, 95% CI 2.80, 4.11, *P* ≤ 0.001) and just culture (OR = 2.65, 95% CI 1.97, 3.56, *P* ≤ 0.001) were still strong predictors of approaching PPSP. Both objective work environment factors maintained strong associations with approaching PPSP. Regarding the individual factors, symptoms of depression (OR = 0.62, 95% CI 0.50, 0.76, *P* ≤ 0.001) and anxiety (OR = 0.66, 95% CI 0.54, 0.80, *P* ≤ .001) still had strong negative associations with approaching PPSP, while none of the demographic factors were significant.

## Discussion

The aim was to investigate what promotes and prevents pilots from contacting PPSP, and to examine the prevalence of possible cases of depression and anxiety disorders among commercial pilots in Europe. Among the psychosocial work environment factors, just culture, support from manager, support from colleagues, workplace satisfaction, and whether one would approach the employer with mental health issues were significant in predicting pilots’ inclination to approach PPSP. In addition, the objective work environment factors, type of employment and minimum guaranteed pay, were significant predictors. More symptoms of depression or anxiety were associated with being less inclined to approach PPSP. In the present sample, the prevalence of possible cases of depression or anxiety disorder was 18% for depression and 23% for anxiety. These numbers are close to the prevalence in the general population, which are 23% and 21%, respectively [29].

Pilots perceiving the organization’s safety culture as just, in which organizations encourage reporting of safety-related events, and accept errors as a part of human nature, appear to be more inclined to approach PPSP. Organizations in which the safety culture is perceived as unjust should be concerned as these results not only indicate that pilots avoid contacting PPSP, but it has also been shown that pilots working in poor safety climates are more prone to mental health issues [30]. In addition, it could be important for operators to routinely measure how pilots perceive their psychosocial work environment, as such measurements may identify contextual barriers to pilots’ mental health help-seeking.

Pilots with atypical types of employment appear to be significantly less likely to approach PPSP if needed. Nevertheless, atypical employment types remain common, especially among European low-cost carriers [7]. Furthermore, pilots with a minimum guaranteed pay regardless of hours flown, that is, having a fixed monthly salary, appear to be more inclined to approach PPSP. This underscores the importance of objective work environment factors in relation to pilots’ inclination to approach peer support. A consequence of this may be that airlines in which pilots have atypical types of contracts, or lack a guaranteed minimum pay, have an increased risk of pilots operating flights with undisclosed mental health issues. To mitigate the flight safety risks thereof, a good practice may be to primarily employ pilots with direct employment and permanent contracts, as well as compensate pilots with a fixed monthly pay.

Symptoms of depression or anxiety disorders also appear to influence pilots’ inclination to approach PPSP in a concerning direction. Pilots scoring 8 or above on HADS-D and HADS-A were significantly less likely to answer that they would approach PPSP. These are troubling findings, as one of the primary targets of PPSP is to attract pilots with mental health issues. In spite of regulations that disqualify pilots with mental health issues from flight duties [3], the results from this study suggest that there may be a significant proportion of active pilots with clinically relevant symptoms of depression or anxiety disorders. However, it cannot be ruled out that pilots with mental health issues receive support elsewhere and therefore have unfavourable attitudes towards PPSP. The present study is, to the authors’ knowledge, the first to investigate to which extent organizational and individual factors are involved in approaching PPSP among pilots. Strengths of the study include the large and wide selection of pilots working all across Europe, enhancing the generalizability of the findings. Participation in the survey was anonymous, which increased the conditions for honest responses, especially in providing statements of mental health [31]. Other strengths include the low levels of missing data in the statistical analyses. Although the proportion of missing data was small, the completion rate of the survey was only 63%. The open survey design has complicated the determination of the characteristics of drop-outs and non-responses, affecting the representativeness of the sample. As the survey was extensive and required substantive effort to complete, it is possible that mental health issues were overrepresented among drop-outs and non-responses. Other limitations include the cross-sectional study design limiting the possibility to make causal inferences and the fact that the dependent variable was a hypothetical scenario.

Research on peer support in the context of the aviation industry is still limited. There is a great need for research on the effectiveness of PPSP regarding pilots’ mental health. Although PPSP are widely implemented within the aviation industry and access is mandatory for commercial pilots in Europe, there is still no evidence of preventive effects of PPSP on pilots’ mental health. This makes it difficult to determine if PPSP is the right way to tackle the probable underreporting of mental health issues among pilots. To mitigate some of the drawbacks of using a hypothetical scenario as the dependent variable, future research on peer support should include objective measures, such as statistics on how often peer support is contacted. There is also a need for longitudinal study designs in order to investigate causal relationships.

This study provides new insights into which factors promote and prevent pilots from approaching PPSP, as well as new estimates of the prevalence of possible depression and anxiety disorders among pilots. The impact of the organizational factors constitutes important knowledge for EASA and national aviation authorities, in ensuring that pilots’ working conditions are such that pilots dare to approach PPSP. Further, the findings could assist operators in developing measures to facilitate pilots’ help-seeking. We also found that pilots with clinically relevant symptoms of depression and anxiety are less likely to seek help from PPSP. Although the data cannot rule out support from other sources as reasons for those unfavourable attitudes, Pilot Peer Support in its current form appears to be an insufficient means to facilitate pilots’ mental health help-seeking. Nevertheless, PPSP may have an important preventive purpose that this study cannot establish. Efforts aimed at getting more pilots to seek help from peer support when needed, and ensuring that the help is adequate, could be important steps in the future development of PPSP. Consequently, such measures could improve mental health among pilots, and thereby flight safety.
